# Supplementary material for: Animal Welfare Problems in Sheep Farming: A Current Overview for Germany Based on Surveys of Veterinary Offices and the Evaluation of Publicly Accessible Court Cases
Source: Animals (Basel). 2025 Jul 17;15(14):2116. doi: 10.3390/ani15142116 (PMC12291943; doi:10.3390/ani15142116)
Supplement: Supplementary file 1 [file animals-15-02116-s001.zip › animals-3713143-supplementary.pdf]

animals

MDPI

Document S1: Questionnaire sent to the veterinary offices

Questionnaire animal welfare cases sheep

* Question type: EA (single choice), MA (multiple choice), FT (free text)

General

1. In which federal state is the veterinary office where you work located? (EA*)

☐ Baden-Württemberg

☐ Bavaria

☐ Berlin

☐ Brandenburg

☐ Bremen

☐ Hamburg

☐ Hesse

☐ Mecklenburg-Western Pomerania

☐ Lower Saxony

☐ North Rhine-Westphalia

☐ Rhineland-Palatinate

☐ Saarland

☐ Saxony

☐ Saxony-Anhalt

☐ Schleswig-Holstein

☐ Thuringia

2. How many sheep farms and sheep are currently registered with you? (FT)

Companies	Number of animals
Free text	Free text

Copyright: © 2025 by the authors. Licensee MDPI, Basel, Switzerland. This article is an open access article distributed under the terms and conditions of the Creative Commons Attribution (CC BY) license (https://creativecommons.org/licenses/by/4.0/).

3. Please indicate the frequency of different company sizes in your area of responsibility. (MA)

A) Sheep farms with **up to 10** animals

☐ < 25% ☐ 25–50% ☐ 50–75% ☐ 75–100%

B) Sheep farms with **up to 50** animals

☐ < 25% ☐ 25–50% ☐ 50–75% ☐ 75–100%

C) Sheep farms with **up to 100** animals

☐ < 25% ☐ 25–50% ☐ 50–75% ☐ 75–100%

D) Sheep farms with **up to 500** animals

☐ < 25% ☐ 25–50% ☐ 50–75% ☐ 75–100%

E) Sheep farms with **up to 1,000** animals

☐ < 25% ☐ 25–50% ☐ 50–75% ☐ 75–100%

F) Sheep farms with **over 1,000** animals

☐ < 25% ☐ 25–50% ☐ 50–75% ☐ 75–100%

Reports/notifications of suspected animal welfare violations in sheep

4. How many animal welfare cases involving sheep have been reported to you on average per year in the last 5 years? (EA)

☐ <5 ☐ 5–10 ☐ 10–15 ☐ 15–20 ☐ >20

5. What are these animal welfare complaints on sheep farms and what is their frequency? Please indicate the frequency in percent. (MA and FT)

a) Housing/accommodation

☐ No weather protection (percentage: free text)
☐ Stocking density too high (percentage: free text)
☐ Free-range animals (percentage: free text)
☐ Other: Free text (percentage: free text)

b) Catering

☐ No water (percentage: free text)
☐ No grass (percentage: free text)
☐ No supplementary feeding (hay) (percentage: free text)
☐ No salt licks/mineral feed (percentage: free text)
☐ Other: Free text (percentage: free text)

c) Health

☐ Lameness (percentage: free text)
☐ Downer sheep (percentage: free text)
☐ Diarrhea (percentage: free text)
☐ Fly maggot infestation (percentage: free text)
☐ Cough/nasal discharge (percentage: free text)
☐ Wool damage/skin changes (percentage: free text)
☐ Obvious wounds (percentage: free text)
☐ High animal losses (percentage: free text)
☐ Other: Free text (percentage: free text)

d) Lambing
☐ Abortions (percentage: free text)
☐ Dead lambs (percentage: free text)
☐ Screaming/hungry lambs (percentage: free text))
☐ Other: Free text (percentage: free text)

e) Management
☐ Failure to shear (especially in summer) (percentage: free text)
☐ Inadequate hoof care (percentage: free text)
☐ Problems with livestock guarding dogs (percentage: free text)
☐ Other: Free text (percentage: free text)

f) Other: Free text (percentage: free text)

6. Do animal welfare complaints on sheep farms increase at certain times of the year? (EA)
☐ Spring (Mar–May) ☐ Summer (Jun–Aug)
☐ Fall (Sep–Nov) ☐ Winter (Dec–Feb)
☐ No

Follow-up and progression of sheep welfare cases

7. How often do reporters misjudge animal welfare violations on sheep farms? (EA)
☐ < 25% ☐ 25–50% ☐ 50–75% ☐ 75–100%

8. How often is an out-of-court settlement possible in animal welfare cases on sheep farms? (EA)
☐ < 25% ☐ 25–50% ☐ 50–75% ☐ 75–100%

9. How often do animal welfare cases on sheep farms result in legal proceedings? (EA)
☐ < 25% ☐ 25–50% ☐ 50–75% ☐ 75–100%

10. How often are animal removals enforced in animal welfare cases on sheep farms? (EA)
☐ < 25% ☐ 25–50% ☐ 50–75% ☐ 75–100%

11. How often are sheep farms banned from keeping animals following animal welfare complaints in your area of responsibility? (EA)
☐ < 25% ☐ 25–50% ☐ 50–75% ☐ 75–100%

12. How often are animal welfare complaints in your area of responsibility followed by a ban on sheep farms? (EA)

    ☐ < 25% ☐ 25–50% ☐ 50–75% ☐ 75–100%

13. A) How often do CC-relevant violations occur on the inspected sheep farms? (EA)

    ☐ < 25% ☐ 25–50% ☐ 50–75% ☐ 75–100%

    B) Which CC-relevant violations are involved? (FT)

    Free text

14. How many sheep farms in your area of responsibility properly identify their animals with ear tags? (EA)

    ☐ < 25% ☐ 25–50% ☐ 50–75% ☐ 75–100%

15. How many registered farms are run by professional shepherds? (EA)

    ☐ < 25% ☐ 25–50% ☐ 50–75% ☐ 75–100%

16. What level of training do the defendants have? (EA; FT in each case)

    a. Master shepherd:

       ☐ < 25% ☐ 25–50% ☐ 50–75% ☐ 75–100%

    b. Journeyman shepherd:

       ☐ < 25% ☐ 25–50% ☐ 50–75% ☐ 75–100%

    c. Agricultural training:

       ☐ < 25% ☐ 25–50% ☐ 50–75% ☐ 75–100%

    d. Layman:

       ☐ < 25% ☐ 25–50% ☐ 50–75% ☐ 75–100%

    e. Sheep farming for several years:

       ☐ < 25% ☐ 25–50% ☐ 50–75% ☐ 75–100%

    f. Sheep farming over several generations:

       ☐ < 25% ☐ 25–50% ☐ 50–75% ☐ 75–100%

    g. Other, please specify: Free text

       ☐ < 25% ☐ 25–50% ☐ 50–75% ☐ 75–100%

17. How often do repeat cases occur in the same company? (EA)

    ☐ < 25% ☐ 25–50% ☐ 50–75% ☐ 75–100%

18. Do livestock owners show understanding after being informed by the veterinary office of immediate animal welfare violations on their own farm? (EA)

    Yes☐ < 25% ☐ 25–50% ☐ 50–75% ☐ 75–100%

    No☐ < 25% ☐ 25–50% ☐ 50–75% ☐ 75–100%

19. Are most of the sheep on farms that are reported/reported due to suspected animal welfare violations docked by the tail? (EA)

    ☐ Yes ☐ No ☐ Not known

20. Has an increased incidence of diarrhea or fly maggot infestation been reported in undocked sheep? (EA)

    ☐ Yes, diarrhea ☐ Yes, fly maggot infestation ☐ No, neither

Killing of sheep by livestock owners

21. How often is improper killing of animals observed? (EA)

    ☐ < 25% ☐ 25–50% ☐ 50–75% ☐ 75–100%

22. What problems with regard to inappropriate killing of sheep occur and are complaints made? Please indicate the frequency in each case. (MA and FT)

    ☐ Lack of knowledge and skills of pet owners in private households (Percentage: free text)

    ☐ No certificate of competence available for commercial use (percentage: free text)

    ☐ Killing of animals without reasonable cause (percentage: free text)

    ☐ Other: Free text

Transporting sheep

23. How often do problems occur when transporting sheep?

    ☐ < 25% ☐ 25–50% ☐ 50–75% ☐ 75–100%

24. What problems are involved in animal welfare violations on sheep farms during animal transportation and how often do they occur? Please indicate the frequency in percent. (MA and FT)

    a) Non-transportable sheep

        ☐ Non-walking or non-standing sheep (percentage: free text)
        ☐ Sheep with large wounds (percentage: free text)
        ☐ Female sheep in the last tenth of pregnancy or up to 7 days after lambing (percentage: free text)
        ☐ Lambs less than one week old (percentage: free text)
        ☐ Other: Free text (percentage: free text)

    b) Defects in transport vehicle/trailer

        ☐ Too high stocking density on transporters (percentage: free text)
        ☐ Risk of injury to the animals (percentage: free text)
        ☐ Danger of sheep falling out (percentage: free text)
        ☐ Floor not resistant to footfall (percentage: free text)
        ☐ Deficiencies with regard to ventilation or fresh air supply (percentage: free text)
        ☐ Other: Free text (percentage: free text)

Conclusion

25. The number of animal welfare cases on sheep farms per year has increased in the last 5 years... (EA)
    ☐ ... decreased.
    ☐ ... remained more or less the same.
    ☐ ... increased.

26. Have you noticed any other animal welfare problems on sheep farms or do you have any further comments for us on this subject? (FT)
    Free text

Disclaimer/Publisher's Note: The statements, opinions and data contained in all publications are solely those of the individual author(s) and contributor(s) and not of MDPI and/or the editor(s). MDPI and/or the editor(s) disclaim responsibility for any injury to people or property resulting from any ideas, methods, instructions or products referred to in the content.
